# Factors associated with extrajudicial arrest for syringe possession: results of a department-wide survey of municipal police in Tijuana, Mexico

**DOI:** 10.1186/s12914-018-0175-1

**Published:** 2018-09-15

**Authors:** Mario Morales, Claudia Rafful, Tommi L. Gaines, Javier A. Cepeda, Daniela Abramovitz, Irina Artamonova, Pieter Baker, Erika Clairgue, Maria Luisa Mittal, Teresita Rocha-Jimenez, Jaime Arredondo, Thomas Kerr, Arnulfo Bañuelos, Steffanie A. Strathdee, Leo Beletsky

**Affiliations:** 1 Division of Infectious Diseases and Global Public Health, School of Medicine, University of California San Diego, 9500 Gilman Dr., La Jolla, San Diego, California 92093 USA; 2 Centre for Urban Health Solutions, St. Michael’s Hospital, Toronto, Canada; 3 School of Medicine, Universidad Xochicalco, 4850 Calle Rampa Yumalinda, Chapultepec Alamar, 22110 Tijuana, Baja California Mexico; 4 School of Public Health, San Diego State University, 5500 Campanile Dr, San Diego, California 92182 USA; 5 Center of Excellence in HIV/AIDS, University of British Columbia, 2206 East Mall, Vancouver, BC V6T 1Z3 Canada; 6 Department of Planning and Special Projects, Secretaría de Seguridad Pública Municipal, 2141 Blvd Cuauhtémoc Sur y Río Suchiate, 22015 Tijuana, Mexico; 7 Health in Justice Action Lab, School of Law and Bouvé College of Health Sciences, Northeastern University, Boston, 02115 USA

**Keywords:** Persons who inject drug, Drug policy, Justice, Human rights, Police, Arrests, Syringe possession

## Abstract

**Background:**

Mexican law permits syringe purchase and possession without prescription. Nonetheless, people who inject drugs (PWID) frequently report arrest for syringe possession. Extrajudicial arrests not only violate human rights, but also significantly increase the risk of blood-borne infection transmission and other health harms among PWID and police personnel. To better understand how police practices contribute to the PWID risk environment, prior research has primarily examined drug user perspectives and experiences. This study focuses on municipal police officers (MPOs) in Tijuana, Mexico to identify factors associated with self-reported arrests for syringe possession.

**Methods:**

Participants were active police officers aged ≥18 years, who completed a self-administered questionnaire on knowledge, attitudes and behaviors related to occupational safety, drug laws, and harm reduction strategies. Univariable and multivariable logistic regression was used to identify correlates of recent syringe possession arrest.

**Results:**

Among 1044 MPOs, nearly half (47.9%) reported always/sometimes making arrests for syringe possession (previous 6mo). Factors independently associated with more frequent arrest included being male (Adjusted Odds Ratio [AOR] = 1.62; 95% Confidence Interval [95% CI] =1.04–2.52; working in a district along Tijuana River Canal (where PWID congregate) (AOR = 2.85; 95%CI = 2.16–3.77); having recently experienced a physical altercation with PWID (AOR = 2.83; 95% CI = 2.15–3.74); and having recently referred PWID to social and health services (AOR = 1.97; 95% CI = 1.48–2.61). Conversely, odds were significantly lower among officers reporting knowing that syringe possession is legal (AOR = 0.61; 95% CI = 0.46–0.82).

**Conclusions:**

Police and related criminal justice stakeholders (e.g., municipal judges, prosecutors) play a key role in shaping PWID risk environment. Findings highlight the urgent need for structural interventions to reduce extra-judicial syringe possession arrests. Police training, increasing gender and other forms of diversity, and policy reforms at various governmental and institutional levels are necessary to reduce police occupational risks, improve knowledge of drug laws, and facilitate harm reduction strategies that promote human rights and community health.

## Background

Restrictive laws and aggressive police enforcement of drug and syringe possession are pervasive risk factors for HIV and other blood-borne infections among people who inject drugs (PWID) [1–20]. Worldwide, law enforcement has negatively impacted PWID willingness to purchase and carry sterile syringes, avoidance of sharing syringes and shooting galleries, and utilization of syringe exchange programs [21–35]. Police confiscation of both used and unused syringes negatively influences the way PWID consume drugs [4, 8, 10, 13–17, 23, 36–48]. In addition to the negative health outcomes of incarceration in terms of infectious disease transmission, overdose, and structural vulnerability risk [49, 50], arrests for syringe possession constitute a human rights and global health concern that may cause immediate and downstream harm to PWID.

Police encounters with PWID involving syringes may also have a negative health impact on police officers [5, 6, 36, 51–57]. In 1996, among 803 participants in the San Diego Police Department, 29.7% had at least one needle stick injury (NSI) ever, of which 27.7% had had two or more; only 39.2% sought medical attention for the NSI [51]. In Baltimore, between 2010 and 2012, 8% of police officers reported lifetime prevalence of NSI [36] and 4% among officers in North Carolina between 2010 and 2012 [55]. Municipal police officers (MPOs) in Tijuana, Mexico, reported a higher prevalence of NSI in 2014 (14.3%) [56]. High rates of NSI put Tijuana MPOs at an elevated risk of blood-borne infections such as HIV, HCV and HBV. The potential for HCV transmission is especially high due to the elevated antibody seroprevalence (96%) among PWID in Tijuana [58].

Police knowledge, attitudes, and behaviors towards PWID are potentially modifiable factors that are related to arrest for syringe possession. Previous research suggests these factors vary and may often be detrimental and antithetical to human rights [2, 5, 6, 36, 38, 59–61]. Police officers in Togliatti (Russia) and Tijuana stopped and frisked PWID because they considered them potential crime suspects [2, 62]. In Rhode Island, police officers (U.S.) were misinformed about the syringe possession law, and those who knew that it was legal to carry syringes continued to treat syringes as contraband [5]. Before receipt of a training on occupational safety, legal knowledge, and harm reduction strategies, half of Rhode Island’s police officers who were trained reported that syringe access increased drug use (51%) and police NSI (58%) [6]. However, Kyrgyz police officers who received a similar (though more lengthy) training as in Rhode Island, were significantly more likely to have improved their occupational safety knowledge, syringe confiscation avoidance, and supporting referrals to harm reduction organizations [60].

Laws allowing syringe possession or acquisition without a prescription are a noted structural intervention to improve community health. However, extrajudicial syringe possession arrests continue even in context of syringe legality. Police knowledge of the law may be a key factor shaping syringe possession arrests. In Mexico, the level of legal knowledge on syringe possession is low among police and among PWID. Previously, we found that only approximately half (56%) of MPOs in Tijuana correctly reported that suspects can possess unlimited numbers of syringes under the law of Baja California [61] and 49% of MPOs reported always/sometimes confiscating syringes in the last 6 months [47]. The discrepancy between black letter law and its understanding was even more pronounced among PWID, where 86% of the participants reported incorrect knowledge of the syringe possession law [38].

In Tijuana, Mexico, the gap between syringes’ formal legal standing and their status under the law on the streets has dire public health implications. HIV prevalence there is triple the national average (0.9% vs. 0.3%) [44], and transmission remains concentrated among high risk populations [63]. Syringe confiscations among PWID are concentrated in the Centro district [47] and along the Tijuana River Canal, an open-air water artery where PWID congregate and consume drugs [37]. Reports of syringe confiscations in the city have been independently associated with PWID risk behaviors such as receptive syringe sharing [16], seeking assistance to inject [17], using high-dead space syringes (which are more likely to transmit blood-borne infections) [64], injecting in shooting galleries [23], and a higher overall prevalence of HIV infection [39, 65].

Despite the critical role of these encounters in shaping health risk, no previous research (to our knowledge) has elucidated factors associated with syringe possession arrests from the perspective of police officers rather than PWID. In general, police officers have poor knowledge of occupational safety related to NSI, syringe possession laws and harm reduction strategies, and also often hold negative attitudes towards PWID, decriminalization and syringe access initiatives [5, 6, 60]. To better understand what drives these detrimental practices, we aimed to identify factors associated with self-reported syringe possession arrests in a large sample of MPOs. We hypothesized that negative experiences with PWID, not referring PWID to social and health services, and inadequate knowledge of the syringe possession law in Mexico would be positively and significantly associated with arrests for syringe possession.

## Methods

This analysis was undertaken as part of the *Escudo* (Shield) project, described in detail elsewhere [56, 61, 66]. From February 2015 to May 2016, 85% of the municipal police force in Tijuana (over 1800 participants) was trained on: [1] occupational safety related to interactions with PWID and blood-borne infections, [2] Mexican drug laws, and [3] harm reduction strategies. Inclusion criteria were: ≥18 years of age, being an active MPO, and willing and able to provide consent. To focus on participants who would be expected to interact with PWID and engage in drug law enforcement, we excluded top police officials and officers engaged in administrative, or other non-street enforcement duties. Baseline surveys were self-administered prior to the *Escudo* training and data were collected on participants’ knowledge, attitudes and behaviors towards the three training topics. The study protocol was approved by the Human Research Protections Program of the University of California, San Diego, and by the Institutional Review Board at Universidad Xochicalco, Tijuana.

### Measures

The outcome, syringe possession arrests, was measured by the item: *“In the last 6 months, how often have you arrested someone for syringe possession?”.* This variable was self-reported and measured on a 5-point Likert scale: “always,” “sometimes,” “rarely,” “never,” and “do not know.” The outcome was dichotomized to always/sometimes, and never/rarely in order to distinguish the frequency of arrests.

Independent variables included sociodemographic characteristics (i.e., sex, age, education, and marital status), policing characteristics (i.e., years in law enforcement, ever been stuck with a needle, and district of service), attitudes towards drug users (i.e., PWID do not care about health, addiction is a disease, and PWID do not deserve to be treated as other people), interactions with PWID (i.e., recent referral of PWID to social and health services and recent physical altercation), and knowledge of the syringe possession law in Baja California.

We generated an ordinal variable to differentiate the number of times that an MPO had ever been stuck with a needle on duty: 0, 1 and 2 or more. The current district of service variable describes the physical location where MPOs were commissioned at the time of the survey. This binary variable differentiates police districts according to their proximity to the Tijuana River Canal: along the Canal (Centro, La Mesa and Mesa de Otay districts) or elsewhere (all other eight districts).

Attitudes towards drug use included three items: “*People addicted to drugs do not care about their health,*” “*Drug addiction is a disease,*” and “*Drug users do not deserve to be treated as other people.*” The first two variables were measured on a 4-point Likert scale (agree, neither agree nor disagree, disagree and do not know). The last variable was measured on a 6-point Likert scale (strongly agree, agree, neither agree nor disagree, disagree, strongly disagree and do not know) and was trichotomized to strongly agree/agree, neither agree nor disagree, and strongly disagree/disagree.

Interactions with PWID were measured by the items: *“In the last 6 months, how many times have you referred drug users to social or health programs?”,* and *“In the last 6 months, how often have you had a physical altercation with a drug user suspect?”.* Both variables were measured on a 5-point Likert scale (always, sometimes, rarely, never and do not know) and were dichotomized for the analysis: always/sometimes and never/rarely.

Knowledge of the law of syringe possession indicated whether an MPO was aware that syringe possession is legal in Baja California. It was measured by posing the following statement: “*Please circle the selection that accurately describes what a suspect can currently possess under the law of Baja California*” (none, 2, 5, 15, as many [syringes] as they want, or do not know). Knowledge was dichotomized to “yes” (if the police officers reported that it is legal to carry at least one syringe), and “no” (if they reported that it is illegal to carry any syringe).

### Statistical analysis

The analysis used data from the baseline assessment. Those who reported arresting always/sometimes for syringe possession in the last 6 months (arrestors) were compared to those who reported never/rarely conducting such arrests (non-arrestors) with respect to selected sociodemographic, knowledge and behavioral characteristics by calculating descriptive statistics. Chi-square and Mann-Whitney tests were used for the comparisons that involved categorical and continuous variables, respectively. Univariable and multivariable logistic regression analyses were conducted to identify characteristics associated with syringe possession arrests. The multivariable logistic regression model was built by entering significant (*p* < 0.10) variables from the univariable analyses using a forward stepwise procedure. The log likelihood test statistic was used to assess model fit and to compare nested models. Potential confounders were considered for inclusion in the multivariable model. Multicollinearity was assessed and ruled out upon obtaining appropriate values for the variance inflation factors and largest condition indices.

## Results

A total of 1751 participants were enrolled and completed the *Escudo* baseline data collection, but 707 MPOs were excluded because they were either higher rank officers, assigned to non-patrol duties, or in service in more than one district. In the resulting sample (*N* = 1044; Table 1), median age was 37 and median years working in law enforcement agencies was 10.5 across groups. Most participants were male (88.5%), married (77.2%) and had at least a high school education (79.1%). Finally, almost half of the participants (47.9%) reported always/sometimes arresting people for syringe possession in the last 6 months.

**Table 1:** Descriptive statistics of municipal police officers by arrest for syringe possession in last 6 months (*N* = 1044)^a^

	Non-arrestor		Arrestor				
	*n* = 544		*n* = 500				
	n/median	%/ IQR	n/median	%/ IQR	Total	%	*p*-value
*Sociodemographic*							
Sex							
Men	471	51.0	452	49.0	923	88.5	**0.064**
Women	72	60.0	48	40.0	120	11.5	
Age	37	32–43	37	32–43	1041	100	0.984
Education							
<High school	105	52.5	95	47.5	200	20.9	0.947
= High School	302	51.6	283	48.4	585	61.1	
>High school	91	52.9	81	47.1	172	18.0	
Marital status							
Single/Never married	70	59.3	48	40.7	118	15.9	0.230
Married/Common law	301	52.4	273	47.6	574	77.2	
Separated/Divorced/Widowed	24	46.2	28	53.9	52	7.0	
*Policing characteristics*							
Years in law enforcement	10.5	6.8–16.3	10.5	5.8–17	975	100	0.991
Number of times ever been stuck by a needle							
0	485	53.5	422	46.5	907	86.9	**0.024**
1	38	48.7	40	51.3	78	7.5	
2 or more	21	35.6	38	64.4	59	5.7	
District of service							
Along Tijuana River Canal	153	37.9	251	62.1	404	38.7	**< 0.001**
Elsewhere	391	61.1	249	38.9	640	61.3	
*Attitudes towards drug use*							
PWUD do not care about their health							
Disagree	45	60.0	30	40.0	75	7.2	0.275
Agree	434	51.9	403	48.2	837	80.3	
Neither agree nor disagree	63	48.5	67	51.5	130	12.5	
Drug addiction is a disease							
Disagree	52	55.3	42	44.7	94	9.1	0.201
Agree	452	51.1	433	48.9	885	86.1	
Neither agree nor disagree	31	63.3	18	36.7	49	4.8	
PWUD do not deserve to be treated as other people							
Disagree	413	53.9	354	46.2	767	74.0	0.148
Agree	74	49.3	76	50.7	150	14.5	
Neither agree nor disagree	54	45.0	66	55.0	120	11.6	
*Interactions with PWUD*							
Referrals of PWUD to social/health programs^a^							
Never/Rarely	379	69.7	246	49.3	625	59.9	**< 0.001**
Always/Sometimes	165	30.3	253	50.7	418	40.1	
Physical altercation with PWUD^a^							
Never/Rarely	356	65.6	199	40.0	555	53.4	**< 0.001**
Always/Sometimes	187	34.4	298	60.0	485	46.6	
*Knowledge of the law of syringe possession*							
Can a person possess syringes under the law of Baja California, Mexico?							
No	156	29.7	180	37.9	336	33.6	**0.006**
Yes	369	70.3	295	62.1	664	66.4	

*IQR* Interquartile range, *PWUD* people who use drugs, ^a^the question refers to the last 6 months, *p* < 0.05 are bolded

Table 1 and Table 2 refer to PWUD, rather than PWID to accurately reflect the Escudo survey as it asks MPOs about attitudes towards PWUD and interactions with PWUD, rather than attitudes towards PWID or interactions with PWID

Relative to the non-arrestors, the arrestor group exhibited a significantly-higher proportion of MPOs who always/sometimes had recent physical altercations with PWID (60% vs. 34.4%, *p* < 0.001), as well as a significantly-higher proportion of recent arrests for syringe possession along the Tijuana River Canal (Centro, La Mesa and Mesa de Otay districts) (62.1% vs. 37.9%, *p* < 0.001). Compared to the non-arrestors, the arrestor group was also more likely to always/sometimes refer PWID to services (50.7% vs. 30.3%, *p* < 0.001) and had a higher proportion of MPOs who have been stuck by a needle two or more times (64.4% vs. 35.6%, *p* = 0.024). Conversely, the non-arrestor groups had a higher proportion of MPOs with the correct understanding of the syringe possession law as compared to the arrestor group (70.3% vs. 62.1%, *p* = 0.006). A marginally-higher proportion of non-arrestors were female relative to the arrestors (60% vs. 40%, *p* = 0.064). Finally, no significant differences were found between the arrestors and non-arrestors with regards to attitudes towards drug use and users.

Table 2 presents the results of the univariable and multivariable logistic regression models. According to the multivariable analysis, MPOs working along the Tijuana River Canal were 2.85 (95% Confidence Interval [CI] = 2.16–3.77) times more likely to always/sometimes arrest somebody for syringe possession, when compared to MPOs working elsewhere. The odds of always/sometimes arresting someone for syringe possession of MPOs who reported a recent physical altercation with PWID were 2.83 (CI = 2.15–3.74) times the corresponding odds of MPOs who did not face such altercation. Additionally, the odds of always/sometimes arresting someone for carrying a syringe were 1.97 (CI = 1.48–2.61) times higher among MPOs who referred PWID to social and health programs as compared to those who did not. Male MPOs were 1.62 (CI = 1.04–2.52) times more likely to always/sometimes arrest somebody for syringe possession, as compared to a female MPOs. Finally, the odds of always/sometimes arresting someone for carrying a syringe among MPOs who knew syringe possession is legal in Baja California were 0.61 (CI = 0.46–0.82) times the corresponding odds of MPOs who did not know the syringe possession law. Despite being significant in the univariable logistic regression model, having been stuck with a needle was not significant in the final model, nor did it affect the significance of other variables in the model.

**Table 2:** Odds of arresting for syringe possession among municipal police officers (*n* = 995)

	OR	95% CI		*p*-value	AOR	95% CI		*p*-value
*Sociodemographic*								
Sex (Ref: Women)								
Men	1.44	0.98	2.12	0.065	**1.62**	1.04	2.52	0.033
Age	1.00	0.98	1.01	0.795				
Education (Ref: <High School)								
= High School	1.04	0.75	1.43	0.831				
> High School	0.98	0.65	1.48	0.938				
Marital status (Ref: Single/Never married)								
Married/Common law	1.32	0.88	1.98	0.173				
Separated/Divorced/Widowed	1.70	0.88	3.28	0.113				
*Policing characteristic*								
Years in law enforcement	1.00	0.98	1.01	0.806				
Number of times ever been stuck by a needle								
1 (Ref: 0)	1.21	0.76	1.92	0.420				
2 or more	**2.08**	1.20	3.60	0.009				
District of service (Ref: Elsewhere)								
Along Tijuana River Canal	**2.58**	1.99	3.33	< 0.001	**2.85**	2.16	3.77	< 0.001
*Attitudes towards drug use*								
PWUD do not care about their health								
Agree (Ref: Disagree)	1.39	0.86	2.25	0.177				
Neither agree nor disagree	1.60	0.90	2.84	0.112				
Drug addiction is a disease								
Agree (Ref: Disagree)	1.19	0.77	1.82	0.434				
Neither agree nor disagree	0.72	0.35	1.46	0.362				
PWUD do not deserve to be treated as other people								
Agree (Ref: Disagree)	1.20	0.84	1.70	0.311				
Neither agree nor disagree	1.43	0.97	2.10	0.072				
*Interactions with PWUD*								
Referrals of PWUD to social/health programs^a^								
All the time/Sometimes (Ref: Never/Rarely)	**2.36**	1.83	3.04	< 0.001	**1.97**	1.48	2.61	< 0.001
Physical altercation with PWUD^a^								
All the time/Sometimes (Ref: Never/Rarely)	**2.85**	2.22	3.67	< 0.001	**2.83**	2.14	3.74	< 0.001
*Knowledge of the law of syringe possession*								
Can a person possess syringes under the law of Baja California, Mexico? (Ref: No)								
Yes	**0.69**	0.53	0.90	0.006	**0.61**	0.46	0.82	< 0.001

*OR* odds ratio, *AOR* adjusted odds ratio, *CI* confidence intervals, *Ref* reference group, ^a^the question refers to the last 6 months, *PWUD* people who use drugs, Significant variables at *p* < 0.05 are bolded

Missing values represented 4.68% of the total observations in the model and 0.97% in the overall sample. “Do not know” answers in the outcome and all the covariates were excluded from the analysis because of their low number of cases (outcome = 0.003%, referral = 0.003%, altercation = 0.006%, and syringe possession law = 0.043%). The model did not present missing values problem considering that the borderline for the missing value analysis on randomness is 5% [67].

## Discussion

In this large study of MPOs in Tijuana, nearly half reported arresting PWID for syringe possession in the past 6 months, despite syringes’ legal status. This proportion is nearly identical to that reported in a study of PWID in Tijuana 10 years earlier [16]. These findings echo data from elsewhere suggesting that liberalizing drug and syringe access policy alone are not sufficient to reduce harm to PWID flowing from policing practices [68]. While knowledge of the syringe law was associated with a lower frequency of arrest for syringe possession, factors like experiences of physical altercation with PWID, having referred PWID to social or health services, patrolling high-intensity drug areas, and being male are also predictive of more frequent syringe possession arrests.

This study’s identification of a significant association between knowledge of syringe possession law and arrests for syringe possession is novel. This link between policy knowledge and street practice adds credence to claims that formal law reform, when operationalized through education programs, may be an effective structural intervention to address officer safety and community health [69]. This finding is particularly relevant considering a previous analysis comparing pre- and post-training surveys with this cohort of MPOs that found significant improvement (*p* < 0.001) in their conceptual knowledge of syringe possession law (from 67 to 96%), defined as the recognition that it is legal to carry one or more syringes [61].

We also found that female MPOs were significantly less likely to report syringe possession arrests compared to male MPOs. Policing is a male-dominated profession and women have been underrepresented in its ranks [70]. This study highlights the role of female MPOs as vital to reducing occupational risks and promoting harm reduction practices among police [47]. Previous research suggests female police officers possess greater empathy and communication skills, and are less likely to engage in aggressive behavior, such as threats, use of force incidents, and extra-judicial search and arrest [71–73]. Interventions that capitalize upon the positive role of female MPOs could have a multiplier effect by improving relations with people who use drugs and the broader community, while advancing officer occupational safety and morale. Beyond individual-level behavior, female MPOs may exert positive peer pressure or other normative influence on policing ecosystems in ways that suppress overall arrests for syringe possession.

Neighborhoods with high drug using activity have previously been identified as key locations for MPOs encounters with PWID [14, 37, 45]. Our findings are consistent with previous analyses of MPOs that identified a positive association between being stationed in Centro, La Mesa and Mesa de Otay districts (high drug use districts along the Tijuana River Canal) and syringe confiscation [37, 45, 47]. In the face of police abuse and harassment reports by PWID [4, 8, 10, 15, 41, 43], police training on harm reduction strategies and enhanced monitoring of arrests for syringe possession are especially important in high drug use neighborhoods.

Unexpectedly, MPOs who reported more frequent referrals of PWID to social and health services during last 6 months were more likely to arrest for syringe possession in the same period of time; it is not clear whether these actions occurred within the context of the same encounter, however. It is possible that these referrals were made to compulsory treatment facilities as a punishment for aberrant behavior that did not qualify as a violation of criminal law [3], or were part of a zero-tolerance approach advanced by the Tijuana’s Ministry of Public Safety [45]. Between December 2014 and March 2015, a policing program known as *Tijuana Mejora* implemented along the Tijuana River Canal resulted in the displacement of 800 to 1000 people, the majority of whom were involuntary transported to “treatment” centers [74]. Future research should assess what MPOs understand as a referral and whether it is characterized as voluntary or compulsory.

MPOs often present PWID in front of a municipal judge after arresting them for syringe possession [62]. In these instances, municipal judges—not the officers—have the authority to dismiss the arrest and/or divert PWID to social and health services. Thus, future interventions should recognize the critical role of municipal judges and other interactors within the criminal justice system above and beyond MPOs. Regardless of how referrals occur, there are limited services specialized for PWID in Tijuana [75]. Many existing substance use treatment facilities offer only abstinence-based modalities that are not evidence-based and may, in fact, be causing more harm than good [75]. In other settings, police referrals to places like substance use treatment providers or safe injection facilities can be a source of benefit for PWID and an opportunity to align public health and security efforts [76]. It is important to understand the current nature of these referrals and work to improve them, concurrently addressing the quality, acceptability, and affordability of substance use resources available in Tijuana, elsewhere in Mexico, and beyond.

This study has several limitations. As a cross-sectional analysis, we cannot infer causal relationships. Qualitative research may assist in elucidating the temporality of these associations, in addition to future analyses from *Escudo* that will assess these associations longitudinally. As this analysis is based on self-reported data while officers were on duty for mandated occupational training, we cannot assume unbiased reporting. Bias may have been minimized since surveys did not include identifying information and were collected by study staff who were not employed by the police department or the police academy. Additionally, precision of our outcome may be a concern since it was not clear how the four answer options of our dependent variable were understood by the participants because it conflated exposure and frequency. For example, an MPO who stops 40 people and arrests 10 is not differentiated from the MPO who stops 10 people and arrest them all. There may also be differential understanding by the MPOs as to what constitutes a referral (voluntary or compulsory) and what constitutes a physical altercation. Further qualitative research needs to define those differences. In this analysis, we cannot determine the impact of peer pressure on arrests for syringe possession. Finally, considering that policing is not a lone activity, there is a need to further explore this topic in subsequent qualitative research. For instance, MPOs may feel pressure from superiors or peers to meet arrest or other encounter quotas.

Overall, in order to reduce harm to PWID and improve occupational safety among police officers, drug and syringe access policy reform should be coupled with police education programs, police oversight mechanisms and structures, effective referral processes, and scale-up in evidence-based drug treatment resources. High drug use neighborhoods should receive particular attention and the critical role of female police officers should be recognized. Future research and interventions are required to maximize MPOs’ knowledge (on occupational safety, drug laws and harm reduction), improve policing tactics and reduce harm in the context of drug and syringe access policy reform. Efforts are also needed to include training of municipal judges, who are responsible for referring PWID to social and health services.

## Conclusions

Our study highlights the need for structural interventions targeting MPOs and related key stakeholders, such as municipal judges and prosecutors. Knowledge of the syringe possession law is associated with reduced arrests for carrying syringes, but altercations with PWID, referrals to services, patrolling hot spots and being male increases the odds of those arrests. Thus, police training, personnel diversification, and policy reforms at various governmental and institutional levels are necessary to reduce police occupational risks, improve knowledge of drug laws, and facilitate harm reduction strategies that promote human rights and community health.

## Abbreviations

HBV: Hepatitis B virus
HCV: Hepatitis C virus
HIV: Human immunodeficiency virus
MPO: Municipal police officer
NSI: Needlestick injury
PWID: People who inject drugs
